# Prenatal and Postnatal Determinants of Outcome in Neonates with Omphalocele: A 25-Year Single-Center Cohort Study

**DOI:** 10.3390/jcm15103615

**Published:** 2026-05-08

**Authors:** Dina Al Namat, Delia Hînganu, Adrian Romulus Roșca, Ludmila Lozneanu, Elena Țarcă, Nadia Al Namat, Razan Al Namat, Elena Hanganu, Jana Bernic, Marius Valeriu Hînganu

**Affiliations:** 1 Faculty of Medicine, Grigore T Popa University of Medicine and Pharmacy, 700115 Iasi, Romania; dina.rosca-al.namat@umfiasi.ro (D.A.N.); ludmila.lozneanu@umfiasi.ro (L.L.); tarca.elena@umfiasi.ro (E.Ț.); hanganu.elena1@umfiasi.ro (E.H.); marius.hinganu@umfiasi.ro (M.V.H.); 2 ”Saint Mary” Emergency Children Hospital, 700309 Iasi, Romania; 3 Faculty of Medicine, The Lower Danube University, 800008 Galați, Romania; nadia.alnamat@gmail.com; 4 Internal Medicine Department, Faculty of Medicine, Apollonia University, 700511 Iasi, Romania; dr.razan_romania@yahoo.com; 5 Discipline of Pediatric Surgery, “Nicolae Testemitanu” State University of Medicine and Pharmacy, MD-2004 Chisinau, Moldova; jana.bernic@usmf.md

**Keywords:** omphalocele, cardiac anomalies, pulmonary anomalies, neonatal outcomes, abdominal wall defects

## Abstract

**Background:** Omphalocele is a congenital defect of the anterior abdominal wall frequently associated with additional anomalies that substantially influence neonatal outcomes. Cardiovascular and pulmonary abnormalities are among the most clinically relevant factors affecting survival, yet their relative prognostic contribution remains incompletely characterized in smaller regional cohorts. **Methods:** This retrospective observational study included 50 neonates with omphalocele treated at a tertiary pediatric surgery center in Northeastern Romania between 2000 and 2025. Demographic characteristics, associated congenital anomalies, surgical management, and clinical outcomes were analyzed. Comparisons were performed between isolated and non-isolated omphalocele cases and according to the presence of cardiac and pulmonary anomalies. Univariate logistic regression was used to evaluate associations between associated anomalies and mortality. **Results:** Associated congenital anomalies were present in the majority of patients. Cardiac malformations were identified in 68% of cases, pulmonary anomalies in 22%, and combined cardiopulmonary anomalies in 20%. Overall mortality was substantial. Mortality was higher in non-isolated compared with isolated omphalocele, although this difference did not reach statistical significance. Univariate analysis showed that pulmonary anomalies were significantly associated with increased mortality (OR = 4.31, 95% CI: 1.20–15.50, *p* = 0.025), whereas cardiac anomalies alone were not significantly associated with mortality. Combined cardiopulmonary anomalies were associated with an increased mortality risk without reaching statistical significance. **Conclusions:** In this cohort, pulmonary anomalies were strongly associated with increased mortality among neonates with omphalocele. These findings suggest that detailed prenatal and postnatal pulmonary assessment may contribute to improved risk stratification and multidisciplinary management in affected neonates. The results should be interpreted in the context of the study’s retrospective design and limited sample size.

## 1. Introduction

Omphalocele is a congenital malformation of the anterior abdominal wall, involving the externalization of abdominal contents through the umbilical ring, with preservation of a membranous covering [1,2]. It represents one of the most severe abdominal wall anomalies encountered in neonatal surgery and is frequently associated with a wide spectrum of additional congenital malformations [3,4]. The reported incidence of omphalocele varies between 1.5 and 3 per 10,000 live births, with significant geographic and demographic variation [5].

Unlike isolated abdominal wall defects, omphalocele is often part of a complex malformative pattern involving multiple organ systems, most commonly the cardiovascular and respiratory systems [6,7,8]. Cardiac anomalies are reported in up to 50–70% of affected neonates and are considered major determinants of survival [9,10]. Pulmonary abnormalities, including pulmonary hypoplasia and respiratory insufficiency related to thoracoabdominal disproportion, also contribute substantially to morbidity and mortality [11,12].

The presence of associated congenital anomalies has been shown to significantly influence both short- and long-term outcomes in neonates with omphalocele [13,14,15]. Despite advances in prenatal diagnosis, neonatal intensive care, and surgical techniques, mortality remains high in patients with complex associated malformations [16,17,18].

Most existing studies have focused predominantly on cardiac defects or have analyzed associated anomalies as a combined category, with limited attention to the specific contribution of pulmonary involvement and its interaction with cardiac pathology [15,19,20,21]. Consequently, the relative prognostic impact of pulmonary anomalies in neonates with omphalocele remains incompletely defined, particularly in single-center cohorts from Eastern Europe.

The aim of this study was to evaluate the impact of cardiac and pulmonary anomalies, individually and in combination, on clinical outcomes and mortality in neonates with omphalocele treated at a tertiary pediatric surgery center in Northeastern Romania over a 25-year period.

## 2. Materials and Methods

### 2.1. Study Design and Population

This retrospective observational study included neonates diagnosed with omphalocele and treated at a tertiary pediatric surgery center in Northeastern Romania between January 2000 and December 2025. A total of 50 patients were identified and included in the analysis. The diagnosis of omphalocele was established prenatally or postnatally based on clinical examination and imaging findings. Both isolated and non-isolated omphalocele cases were included.

#### Inclusion and Exclusion Criteria

All consecutive neonates diagnosed with omphalocele and treated at our tertiary pediatric surgery center between January 2000 and December 2025 were considered eligible. Both isolated and non-isolated cases were included, including syndromic or chromosomal conditions when documented in the medical records, in order to reflect the clinical spectrum of the disease. Patients were excluded only if key outcome data were unavailable or if records were incomplete regarding associated anomaly status or survival.

### 2.2. Data Collection

Clinical data were obtained retrospectively from hospital medical records. Collected variables included demographic characteristics (sex, gestational age, birth weight), type of omphalocele (isolated or associated), presence and type of associated congenital anomalies (with particular focus on cardiac and pulmonary malformations), surgical management, length of hospitalization, need for intensive care, and survival outcomes.

Gestational age and birth weight were recorded for all patients. Prematurity and low birth weight were common in the cohort. Omphalocele size was classified clinically, including identification of giant defects. These baseline prognostic variables have been incorporated into the Section 3.

Pulmonary anomalies were defined as conditions associated with clinically relevant respiratory compromise, including pulmonary hypoplasia confirmed by imaging and clinical course, persistent pulmonary hypertension of the newborn (PPHN) requiring targeted intensive care management, and severe respiratory insufficiency necessitating prolonged ventilatory support beyond the immediate postoperative period. Structural lung malformations were recorded when documented in imaging reports.

Cardiac anomalies were identified based on echocardiographic assessment and included congenital heart defects such as ventricular septal defects, atrial septal defects, tetralogy of Fallot, complex cyanotic malformations, and other structural abnormalities. Due to the retrospective design and limited sample size, cardiac defects were analysed as a categorical variable (present/absent), and detailed severity stratification was not consistently possible across the full study period.

Mortality was defined as death occurring during the initial hospitalization.

### 2.3. Immunohistochemical Analysis

Sample Collection and Tissue Procurement.

Tissue samples were coded prior to histological, immunohistochemical, and ultrastructural analyses. All evaluations and scoring procedures were performed according to predefined criteria by investigators blinded to individual clinical data.

#### 2.3.1. Tissue Sampling Protocol in Patients Operated for Omphalocele

Specimens were obtained from patients undergoing surgical repair of omphalocele, corresponding to the cohort included in the histological and immunohistochemical analyses. A standardized sampling protocol was applied in all cases. Full-thickness fragments of the anterior abdominal wall, including fascial and muscular components, were obtained at both the supraumbilical and subumbilical margins of the defect. Specimens were immediately oriented, labeled, and preserved in a formaldehyde-based fixative. As the use of external healthy control tissue was not ethically permissible, an intra-patient comparative model was implemented. Paired samples from supraumbilical and subumbilical regions of the same individual served as internal controls, allowing reliable regional comparisons within identical biological and clinical settings.

#### 2.3.2. Surgical Approach and Incision

The surgical management followed widely accepted techniques described in contemporary surgical practice. All procedures were carried out under general anesthesia, using a midline approach adapted to the dimensions and anatomical characteristics of the omphalocele sac.

A median incision extending both supraumbilically and subumbilically was performed, centered on the defect. This incision encompassed the base of the sac and the umbilical remnant, providing adequate exposure of the herniated viscera and the anterior abdominal wall.

The subcutaneous layers were carefully dissected along the incision line, with particular attention to preserving vascular integrity. The omphalocele sac was subsequently opened and resected as required by the surgical repair strategy. Following clear identification of the defect margins and the rectus abdominis muscles, tissue samples were obtained for further analysis.

#### 2.3.3. Tissue Sampling Sites and Technique

Tissue samples were collected from the anterior rectus sheath together with the underlying rectus abdominis muscle at the edges of the abdominal wall defect, focusing on the interface between dysplastic and relatively preserved parietal structures.

In each patient, sampling was systematically performed at two predefined anatomical levels: the supraumbilical and the subumbilical margins of the defect.

At each site, small full-thickness fragments of the anterior rectus sheath were excised.

#### 2.3.4. IHC Protocol

The immunohistochemical protocols followed standardized procedures widely used in histological studies, ensuring reproducibility and comparability of results. Formalin-fixed tissue samples were processed through paraffin embedding, followed by sectioning and subsequent rehydration. The obtained sections were then incubated in phosphate-buffered saline (PBS) prior to immunohistochemical staining.

Endogenous peroxidase activity was blocked using 3% hydrogen peroxide, followed by incubation with Anti-Pro-Collagen III Alpha 1 antibody (clone RM1028, Abcam, Cambridge, United Kingdom, ab283694; antigen retrieval pH 9; dilution 1:1000; overnight incubation at 4 °C). Detection was performed using a polymer-based detection system (Bond Polymer Refine Detection Kit, Leica Biosystems, Deer Park, IL, United States), and visualization was achieved with 3,3′-diaminobenzidine (DAB).

The sections were incubated with the primary antibodies under controlled conditions (either at room temperature or overnight at 4 °C), after which signal detection was performed using an appropriate detection system (polymer-based or avidin–biotin).

Immunoreactivity was visualized using 3,3′-diaminobenzidine (DAB), and the slides were counterstained with hematoxylin, followed by dehydration and mounting.

Positive and negative controls were included in each staining batch to ensure the validity of the procedure.

#### 2.3.5. Evaluation of Immunohistochemical Staining

Immunohistochemical expression was independently assessed by [one/two] blinded observers using a semi-quantitative scoring system.

Staining intensity was graded on a four-level scale: 0 (negative), 1 (weak), 2 (moderate), and 3 (strong). The proportion of positively stained cells was also recorded.

An H-score was subsequently calculated by combining staining intensity and the percentage of positive cells, resulting in a final score ranging from 0 to 300.

### 2.4. Statistical Analysis

Statistical analysis was performed using standard biomedical statistical methods. Categorical data are presented as absolute counts and proportions, whereas continuous variables are summarized using median values and interquartile ranges (IQR), given their non-normal distribution. Group comparisons were conducted using the chi-square test or Fisher’s exact test for categorical variables and the Mann–Whitney U test for continuous variables.

The relationship between associated congenital anomalies and mortality was assessed by calculating odds ratios (ORs) together with their corresponding 95% confidence intervals (CI). Univariate logistic regression models were applied to estimate the risk of mortality in patients presenting with cardiac anomalies, pulmonary anomalies, and combined cardiopulmonary malformations, using cases with isolated omphalocele as the reference category. Statistical significance was set at a *p*-value < 0.05.

Due to the relatively small number of observed mortality events (n = 21), multivariable logistic regression was not considered appropriate, in order to minimize the risk of model overfitting. In accordance with the widely accepted principle requiring approximately 10 outcome events per predictor variable, the inclusion of multiple covariates would not have provided reliable estimates. Consequently, the analysis was restricted to targeted univariate models focusing on clinically relevant predictors.

Immunohistochemical (IHC) scores were summarized using median values and interquartile ranges (IQR). Group comparisons were performed using the Mann–Whitney U test. A threshold of *p* < 0.05 was used to define statistical significance.

### 2.5. Outcomes

The primary outcome of the study was in-hospital mortality, defined as death occurring during the initial hospitalization period. Secondary descriptive parameters included length of hospitalization and need for intensive care support. Due to variability in retrospective documentation across the 25-year study period, detailed morbidity measures such as duration of mechanical ventilation and long-term follow-up were not consistently available for formal analysis.

### 2.6. Ethical Considerations

All procedures were performed in compliance with the Declaration of Helsinki and were approved by the Institutional Ethics Committee of the “Grigore T. Popa” University of Medicine and Pharmacy, Iași, Romania (approval no. 373/02.01.2024). Informed consent for the use of clinical data was obtained from parents or legal guardians before enrollment.

## 3. Results

### 3.1. Demographic Characteristics

The study included 50 neonates diagnosed with omphalocele. Sex distribution was equal, with 25 male and 25 female patients. Regarding the environment of origin, most patients came from rural areas (n = 44, 88%), while a smaller proportion originated from urban areas (n = 6, 12%).

Gestational age at birth had a median of 38 weeks (IQR 36–39), with 20% of neonates born prematurely (<37 weeks). The median birth weight was 2625 g (IQR 2250–3087.5). Giant omphalocele was identified in 2 cases (4%). These baseline perinatal characteristics are summarized in Table 1.

### 3.2. Associated Congenital Anomalies

Associated congenital anomalies were frequently observed. Cardiac anomalies were the most frequent associated malformations (Table 2). Combined cardiac and pulmonary anomalies were observed in 10 patients (20%).

### 3.3. Mortality and Outcome Analysis

Overall mortality in the cohort was 42% (21/50 patients). The majority of deaths occurred in patients with associated congenital anomalies, accounting for 86% of all mortality cases, whereas only a small proportion of deaths were observed in isolated omphalocele.

Univariate logistic regression analysis identified pulmonary anomalies as significantly associated with increased mortality (OR = 4.31, 95% CI: 1.20–15.50, *p* = 0.025). Combined cardiopulmonary anomalies were associated with higher odds of mortality; however, this association did not reach statistical significance (OR = 3.67, 95% CI: 0.88–15.34, *p* = 0.074). Non-isolated omphalocele alone was not significantly associated with mortality compared to isolated cases (OR = 2.29, 95% CI: 0.52–10.02, *p* = 0.32) (Table 3).

**Figure 1:**
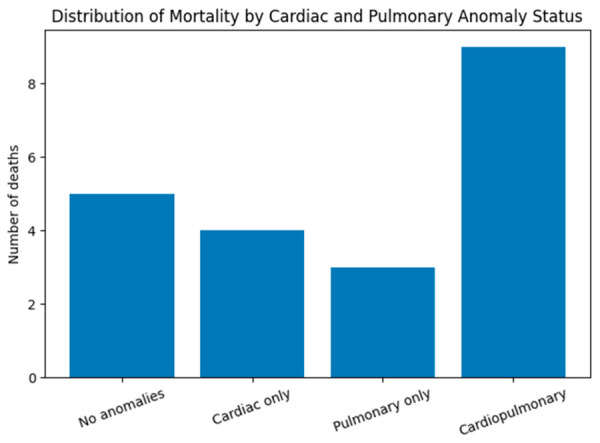
Distribution of mortality according to cardiac and pulmonary anomaly status in neonates with omphalocele. Bars represent the number of deaths (n = 21) stratified into four mutually exclusive categories: no associated anomalies, cardiac anomalies only, pulmonary anomalies only, and combined cardiopulmonary anomalies.

While cardiac anomalies were frequently observed, their isolated presence did not show a statistically significant association with mortality in this cohort. As shown in Figure 1, mortality was most frequent in the combined cardiopulmonary subgroup.

### 3.4. Representative Clinical Cases

To illustrate the clinical heterogeneity and variability in outcomes among patients diagnosed with omphalocele, two representative cases from the present cohort are presented.

**Figure 2:**
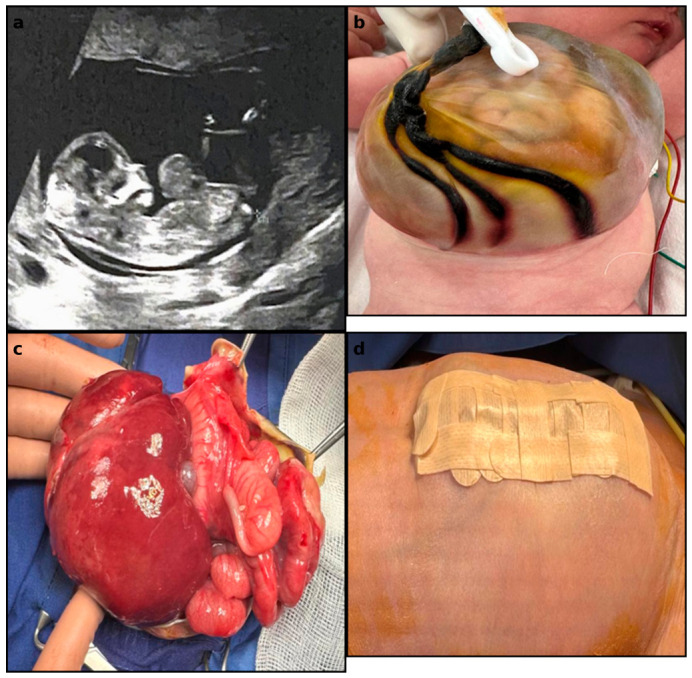
(**a**) Prenatal ultrasound image showing giant omphalocele; (**b**) Postnatal appearance of the omphalocele sac; (**c**) Intraoperative aspect during visceral reduction; (**d**) Postoperative appearance after staged abdominal wall closure.

The first case involved a neonate diagnosed with a giant omphalocele without associated major congenital anomalies (Figure 2). Despite the large size of the abdominal wall defect, postnatal evaluation did not identify significant cardiac or pulmonary malformations. The patient underwent staged surgical management, including gradual reduction in the herniated viscera and delayed abdominal wall closure. Postoperative evolution was favorable, with gradual respiratory stabilization during hospitalization.

**Figure 3:**
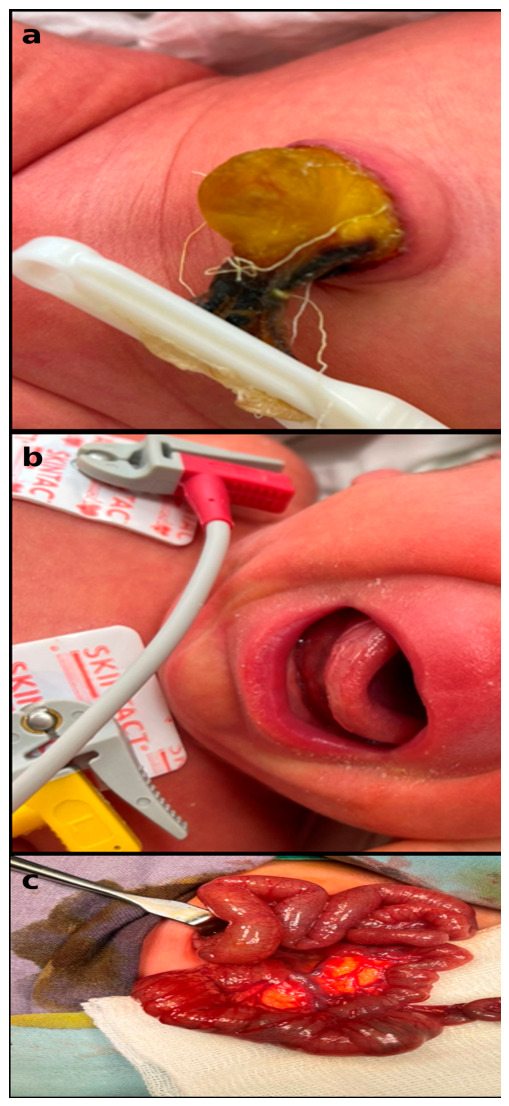
(**a**) Postnatal aspect of the umbilical region showing a small omphalocele; (**b**) Clinical appearance of the neonate during early postnatal period; (**c**) Intraoperative aspect revealing associated intestinal and visceral abnormalities.

The second case represents a neonate with omphalocele associated with severe congenital cardiac anomalies (Figure 3). Prenatal and postnatal assessments revealed complex cardiovascular involvement, which significantly influenced clinical management. Despite surgical intervention and intensive supportive care, the postoperative course was complicated by cardiopulmonary anomalies instability, ultimately resulting in an unfavorable outcome.

The grouping of pulmonary hypoplasia, PPHN, and prolonged ventilatory dependence reflects a functionally defined category of clinically relevant pulmonary compromise, rather than a purely structural classification.

These two cases exemplify the wide spectrum of clinical presentations observed in omphalocele and highlight the prognostic relevance of associated congenital anomalies. No statistically significant association between defect size and mortality was identified in this cohort. This may explain why mortality can also occur in cases with smaller defects, where associated cardiopulmonary abnormalities—particularly pulmonary dysfunction—rather than anatomical size, represent the primary determinant of clinical outcome.

### 3.5. Results of Immunohistochemical Analysis

To explore potential tissue-level mechanisms associated with omphalocele, immunohistochemical (IHC) analyses were performed as an exploratory adjunct to the clinical study.

**Figure 4:**
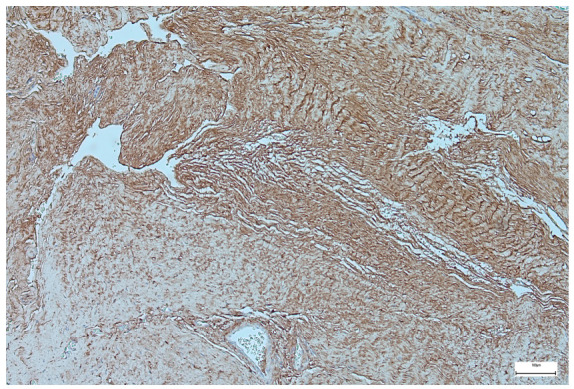
Representative immunohistochemical staining for Pro-Collagen α1 in omphalocele tissue. Moderate-to-strong Pro-Collagen α1 expression with a diffuse fibrillar pattern. Scale bar = 100 μm.

Pro-Collagen α1 expression was consistently observed across analyzed specimens, showing moderate to strong cytoplasmic and extracellular matrix staining, indicative of increased collagen deposition and active fibrotic remodeling within the abdominal wall tissues (Figure 4).

**Figure 5:**
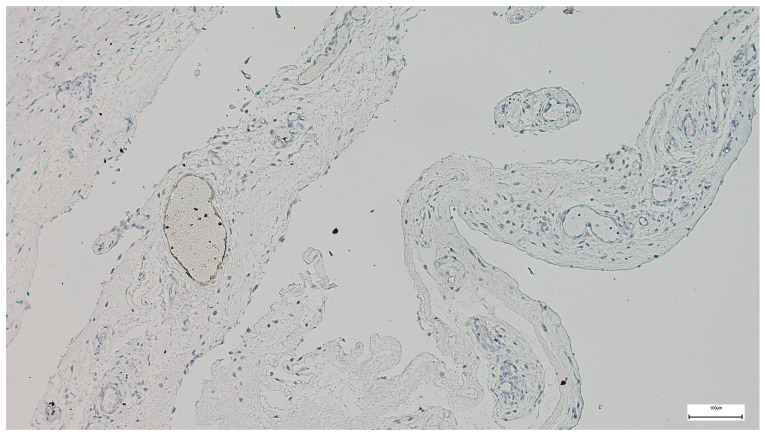
Representative immunohistochemical staining for vascular endothelial growth factor (VEGF) in omphalocele tissue, showing weak to nearly absent immunoreactivity, with minimal cytoplasmic staining and lack of evident angiogenic activity. Scale bar = 100 μm.

In contrast, VEGF expression was weak or nearly absent in most samples, with minimal cytoplasmic staining and no clear evidence of active angiogenic signaling (Figure 5).

These findings suggest a predominance of extracellular matrix remodeling over angiogenic activity in the examined tissues. However, no direct association between IHC patterns and clinical outcomes or the presence of cardiopulmonary anomalies could be established in this cohort.

Given the exploratory nature of this analysis, these observations should be interpreted as hypothesis-generating and may warrant further investigation in larger, prospective studies.

## 4. Discussions

In the present cohort of 50 patients with omphalocele, isolated cases were relatively uncommon, while the majority presented with associated structural or syndromic anomalies. This distribution is consistent with previous large clinical series and systematic reviews, which report that isolated omphalocele represents a minority of cases in current practice [22,23].

Although mortality tended to be higher among non-isolated cases, this association did not reach statistical significance in our cohort. This finding suggests that outcomes are not determined solely by the presence of associated anomalies, but rather by their type and functional impact. Similar observations have been reported by Parata et al., who demonstrated that prognosis is influenced more by the burden and nature of associated anomalies than by defect size alone [24].

In this context, our results highlight the potential importance of pulmonary involvement in the clinical evolution of neonates with omphalocele. While cardiac anomalies were frequently observed, they did not show a statistically significant association with mortality when analyzed independently. In contrast, pulmonary anomalies demonstrated the strongest association with adverse outcomes, suggesting that pulmonary compromise may play a central role in early neonatal instability.

These findings are consistent with clinical experience, where conditions such as pulmonary hypoplasia, persistent pulmonary hypertension, and thoracoabdominal disproportion frequently represent critical determinants of survival. Pulmonary abnormalities may directly impair postnatal adaptation and perioperative management, thereby significantly influencing outcomes even when surgical repair is technically feasible.

The coexistence of cardiopulmonary anomalies likely reflects a broader developmental vulnerability, contributing to a higher-risk phenotype. Previous studies have similarly emphasized the combined impact of cardiac defects and pulmonary morbidity on neonatal survival and postoperative evolution [23,24].

Advances in prenatal imaging have improved the ability to identify high-risk cases. While ultrasound remains the primary diagnostic tool, fetal MRI has been increasingly used for risk stratification. Parameters such as observed-to-expected total fetal lung volume and the degree of liver herniation have been associated with postnatal mortality and the need for staged repair [25]. These metrics may enhance prenatal counseling and guide perinatal management strategies.

These findings in our study revealed structural heterogeneity at the margins of the abdominal wall defect, supporting the concept that omphalocele is associated with intrinsic abnormalities of tissue organization rather than simple displacement of normal structures. These observations provided the rationale for further exploratory tissue-level analysis [26,27].

Immunohistochemical evaluation demonstrated consistent Pro-Collagen α1 expression, suggesting active extracellular matrix remodeling, whereas VEGF expression was minimal. This pattern may indicate a predominance of fibrotic remodeling over angiogenic activity in the examined tissues, aligning with current concepts of abnormal connective tissue development in omphalocele [28,29]. However, no direct correlation between these findings and clinical outcomes or cardiopulmonary anomalies could be established.

These tissue-level observations should therefore be interpreted as exploratory and hypothesis-generating. Their potential relevance for surgical repair and postoperative evolution remains to be clarified. Altered extracellular matrix composition could influence tissue compliance and may contribute to challenges in defect closure or to postoperative complications such as ventral hernia formation [30,31].

Several limitations must be acknowledged. The retrospective single-center design limits generalizability, and the long inclusion period (2000–2025) introduces temporal heterogeneity related to advances in prenatal diagnosis, neonatal intensive care, and surgical management. These evolving practices may have influenced outcomes independently of associated anomalies.

The relatively small sample size and limited number of outcome events precluded robust multivariable analysis. As a result, residual confounding between cardiac and pulmonary anomalies cannot be excluded. Key clinical variables such as gestational age, birth weight, defect size, and syndromic context could not be consistently incorporated into adjusted models due to incomplete data availability.

In addition, detailed information regarding ventilatory strategies, including the use of inhaled nitric oxide, surfactant therapy, and advanced hemodynamic monitoring, was not uniformly available. These factors may significantly influence outcomes and represent an important limitation of the study.

The immunohistochemical component was exploratory and limited in scope, without extensive quantitative analysis or uniform genetic characterization across the study period.

Recent evidence highlights the growing role of fetal MRI in risk stratification of omphalocele, particularly through quantitative assessment of fetal lung development. Parameters such as observed-to-expected lung volume and liver herniation have been shown to correlate with neonatal morbidity and mortality [32,33].

In our cohort, defect size alone did not appear to predict outcome, suggesting that anatomical severity may not fully reflect the underlying physiological risk. This may explain why adverse outcomes can also occur in cases with smaller defects, where associated cardiopulmonary abnormalities—particularly pulmonary dysfunction—play a decisive role. In this context, recent studies have highlighted the value of fetal MRI in refining risk stratification, particularly through quantitative assessment of lung development, such as observed-to-expected lung volume and the degree of liver herniation. These parameters provide a more accurate estimation of functional pulmonary capacity and have been shown to correlate with neonatal respiratory morbidity and survival. Taken together, these findings support the concept that functional impairment, rather than defect size alone, represents the key determinant of prognosis in neonates with omphalocele.

The close correlation between reduced fetal lung volume on MRI and adverse neonatal outcomes underscores the value of fetal MRI in predicting postnatal prognosis. Measuring fetal lung volumes can help clinicians guide perinatal counseling and tailor management strategies accordingly [33,34].

These findings are consistent with our results, which emphasize the prognostic importance of pulmonary involvement. Previous studies have demonstrated that pulmonary hypoplasia and pulmonary hypertension are major determinants of adverse outcomes in omphalocele [35,36].

Larger multicenter studies with standardized data collection and era-adjusted statistical modeling are needed to further clarify the prognostic role of cardiopulmonary anomalies and to validate the findings of this study.

## 5. Conclusions

This study provides a longitudinal characterization of neonates with omphalocele treated in a tertiary pediatric surgery center over a 25-year period. In this cohort, the presence of associated anomalies—particularly pulmonary involvement—was associated with increased mortality, whereas isolated omphalocele showed more favorable outcomes.

These findings support the importance of comprehensive prenatal and postnatal evaluation of cardiopulmonary anomalies in neonates with omphalocele and highlight the need for multidisciplinary management in tertiary care settings. Given the retrospective design and limited cohort size, the results should be interpreted as exploratory. Larger multicenter studies with standardized definitions and multivariable analysis are required to better define prognostic factors and improve risk stratification in affected neonates.

## Figures and Tables

**Table 1:** Baseline perinatal and clinical characteristics of neonates with omphalocele (n = 50). Values are presented as median (interquartile range, IQR) for continuous variables and as number (percentage) for categorical variables. Prematurity was defined as gestational age <37 weeks. Giant omphalocele was defined clinically based on defect size and/or liver herniation documented in operative or neonatal records. Cardiac and pulmonary anomalies were identified based on postnatal echocardiographic and clinical evaluation.

Variable	Value
Gestational age, median (IQR)	38 weeks (36–39)
Prematurity (<37 weeks), n (%)	10 (20%)
Birth weight, median (IQR)	2625 g (2250–3087.5)
Male sex, n (%)	25 (50%)
Rural origin, n (%)	44 (88%)
Giant omphalocele, n (%)	2 (4%)
Cardiac anomalies, n (%)	34 (68%)
Pulmonary anomalies, n (%)	11 (22%)
Mortality, n (%)	21 (42%)

**Table 2:** Distribution of survival and mortality according to the presence of associated anomalies in patients with omphalocele. Mortality was higher in non-isolated cases compared to isolated cases (46.2% vs. 27.3%); however, the difference did not reach statistical significance (Fisher’s exact test, *p* = 0.32).

	Survived	Deceased	Total
Isolated	8	3	11
Non-isolated	21	18	39
Total	29	21	50

**Table 3:** Univariate logistic regression analysis for mortality.

Predictor	OR	95% CI	*p*-Value
Non-isolated omphalocele	2.29	0.52–10.02	0.32
Cardiac anomalies	0.29	0.08–1.03	0.056
Pulmonary anomalies	4.31	1.20–15.50	0.025
Combined cardiopulmonary anomalies	3.67	0.88–15.34	0.074

## Data Availability

The original contributions presented in this study are included in the article. Further inquiries can be directed to the corresponding author.
